# Zeeman splitting via spin-valley-layer coupling in bilayer MoTe_2_

**DOI:** 10.1038/s41467-017-00927-4

**Published:** 2017-10-06

**Authors:** Chongyun Jiang, Fucai Liu, Jorge Cuadra, Zumeng Huang, Ke Li, Abdullah Rasmita, Ajit Srivastava, Zheng Liu, Wei-Bo Gao

**Affiliations:** 1 Division of Physics and Applied Physics, School of Physical and Mathematical Sciences, Nanyang Technological University, Singapore, 637371 Singapore; 2 Center for Programmable Materials, School of Materials Science & Engineering, Nanyang Technological University, 50 Nanyang Avenue, Singapore, 639798 Singapore; 3 Department of Physics, Emory University, Atlanta, GA 30322 USA; 4 The Photonics Institute and Centre for Disruptive Photonic Technologies, Nanyang Technological University, Singapore, 637371 Singapore

## Abstract

Atomically thin monolayer transition metal dichalcogenides possess coupling of spin and valley degrees of freedom. The chirality is locked to identical valleys as a consequence of spin–orbit coupling and inversion symmetry breaking, leading to a valley analog of the Zeeman effect in presence of an out-of-plane magnetic field. Owing to the inversion symmetry in bilayers, the photoluminescence helicity should no longer be locked to the valleys. Here we show that the Zeeman splitting, however, persists in 2H-MoTe_2_ bilayers, as a result of an additional degree of freedom, namely the layer pseudospin, and spin–valley-layer locking. Unlike monolayers, the Zeeman splitting in bilayers occurs without lifting valley degeneracy. The degree of circularly polarized photoluminescence is tuned with magnetic field from −37% to 37%. Our results demonstrate the control of degree of freedom in bilayer with magnetic field, which makes bilayer a promising platform for spin-valley quantum gates based on magnetoelectric effects.

## Abstract (web summary)

Monolayer transition metal dichalcogenides host a valley splitting in magnetic field analogous to the Zeeman effect. Here, the authors report that the Zeeman splitting still persists in bilayers of MoTe_2_ without lifting the valley degeneracy, due to spin–valley-layer coupling.

## Introduction

In monolayer group VI transition metal dichalcogenides (TMDs) such as MoS_2_ and WSe_2_, broken spatial inversion symmetry leads to finite but opposite Berry curvature and magnetic moment in the two valleys^1–3^. Altogether with strong spin–orbit interaction, broken symmetry enables the coupling of spin and valley degrees of freedom, which gives rise to a series of exotic valley effects, such as the valley Hall effect^4, 5^, valley optical selection rule^6–9^, and valley Zeeman splitting^10–15^. In bilayer TMDs, the layers are rotated by 180° with respect to each other, leading to the recovery of inversion symmetry. It is therefore natural to query whether the above-mentioned valley-chirality still persists in bilayer TMDs. When the interlayer coupling is much smaller than the spin–orbit interaction, a bilayer can be regarded as two decoupled monolayers with the layer pseudospin leading to a spin–valley-layer coupling. This can be potentially utilized as a platform for spin–valley quantum gates with magnetic and electric control^16^. To this end, spin-layer locking induced valley Hall effect^17^, spin-polarized bulk bands^18^, valley optical selection rule^19^, and electric control^20^ have been experimentally investigated. In this work, we demonstrate the Zeeman splitting persisting in bilayer 2H-MoTe_2_ due to spin–valley-layer locking by means of polarization-selective magneto-photoluminescence. The circularly polarized photoluminescence of opposite helicity shows spectral splitting in the presence of an out-of-plane magnetic field despite the inversion symmetry of the bilayer system. Our study shows that in bilayer TMDs, the magnetic field has an important role in the toolbox for exploring the rich interplay between real spin and valley, layer pseudospins in bilayer TMDs. The magnetic control, together with electric control as demonstrated previously, pave the way for quantum manipulation of spin, valley, and layer degrees of freedom in bilayer TMDs^16^.

## Results

### Sample characterization

We perform our experiments on 2H-MoTe_2_, which is a layered semiconductor with hexagonal lattice. With decreasing number of layers, the indirect bandgap of bulk MoTe_2_ turns into direct bandgap^21, 22^. Berry curvature and orbital magnetic moments can be studied through the polarization-selective emission of photoluminescence. Monolayer and bilayer 2H-MoTe_2_ have a relatively smaller bandgap among the TMDs and their photoluminescence emission lies in the near infrared range around ~1.1 eV. A reversible structural phase transition between hexagonal and stable monoclinic has been reported in bulk single-crystalline MoTe_2_^23^ and a semiconductor-to-metal electronic phase transition has been demonstrated by thinning down bulk MoTe_2_ or straining the MoTe_2_ thin films^24^. These features make MoTe_2_ a flexible material suitable for valley-based optoelectronic applications.

**Fig. 1:**
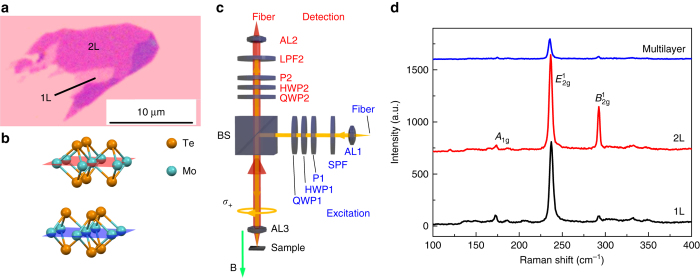
Sample characterization. **a** Optical microscope image of the MoTe_2_ monolayer and bilayer. **b** Crystal structure of a bilayer MoTe_2_. The two layers are rotated in-plane by 180° relative to each other. **c** Optical setup for the polarization-resolved PL spectroscopy. The optical components are: achromatic lenses (AL1-3), polarizers (P1 and P2), half-wave plates (HWP1 and HWP2), quarter-wave plates (QWP1 and QWP2), a short pass filter (SPF), a long pass filter (LPF), and a beam splitter (BS). The sample is placed in a helium bath cryostat with an out-of-plane magnetic field in a Faraday geometry. The green arrow shows a negative magnetic field. **d** Raman spectroscopy of the MoTe_2_ monolayer, bilayer, and multilayer. *A*_1g_, $B_{2{\rm{g}}}^1$, and $E_{2{\rm{g}}}^1$ represent different modes in Raman spectroscopy

An optical image of the studied sample is illustrated in Fig. 1a, where the monolayer (1L) and bilayer (2L) can be easily identified by their optical contrasts. The flakes are mechanically exfoliated using adhesive tapes and then transferred onto a silicon wafer with a 300 nm thick thermally grown SiO_2_. The as-prepared samples are kept under vacuum to prevent oxidation and deliquesce. The crystal structure of a bilayer AB-stacked MoTe_2_ is shown in Fig. 1b. The bilayer has inversion symmetry as compared to monolayers. Monolayer and bilayer MoTe_2_ have exciton energy of ~1.1 eV, which can be experimentally extracted by the photoluminescence (PL) spectroscopy. We utilize a homemade fiber-based confocal microscope setup for the micro-PL experiments (Fig. 1c). We show the details of our experimental setup in the “Methods” section. The excitation and collection polarizations are controlled by a series of polarizers and quarter-wave plates. Below, we refer to co-polarization (cross-polarization) when the quarter-wave plates are configured for the same (opposite) handedness.

To further confirm the number of layers in our sample, we perform Raman spectroscopy of the monolayer, bilayer, and multilayers at room temperature as shown in Fig. 1d. The $B_{2{\rm{g}}}^1$ mode of the 2L is strong with a Raman shift of 292.4 cm^−1^ while that of the 1L and multilayer is very weak. The in-plane mode $E_{2{\rm{g}}}^1$ (out-of-plane mode *A*_1g_) exhibits downshift (upshift) in energy as the number of layer increases. The results agree well with the previous report^25, 26^, confirming the number of layers of the investigated sample. Temperature-dependent exciton and Trion peaks in PL measurements (Supplementary Note 1; Supplementary Fig. 1) also show results consistent with previous reports^21, 22^. The exciton peak shows a linear power dependence, whereas the trion peak shows a sub-linear dependence with ${I_{{\rm{PL}}}} \propto I_{{\rm{ex}}}^{0.8}$, where *I*_PL_ and *I*_ex_ are the intensity of the excitation and photoluminescence, respectively (Supplementary Fig. 2).

### Polarization-resolved magneto-photoluminescence spectroscopy

**Fig. 2:**
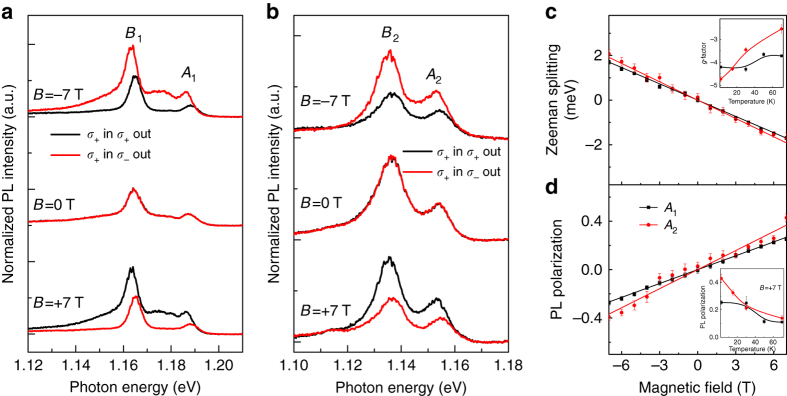
Zeeman splitting and PL polarization in bilayer MoTe_2_. **a**, **b** Off-resonant polarization-resolved PL spectra taken at −7, 0, and +7 T at 2 K with an excitation energy of 1.560 eV (795 nm) in monolayer MoTe_2_ (**a**) for reference and bilayer MoTe_2_ (**b**). The excitation is set to *σ*_+_ circular polarization. The detection polarization is configured to *σ*_+_ (black solid lines) and *σ*_−_ (red solid lines) circular polarization. The PL intensity of the bilayer is one order of magnitude smaller than that in the monolayer due to higher symmetry of the bilayer. Here the PL spectra is normalized by emission maximum for each particular magnetic field and offset for better visualization. **c**, **d** The Zeeman splitting (**c**) and PL polarization (**d**) of the neutral exciton (peak *A*_2_) in bilayer MoTe_2_ (red symbols and lines) vs. magnetic field at 2 K. The inset shows the PL polarization at different temperature at +7 T. The black symbols and lines are results of the monolayer (peak *A*_1_) for reference. The error bars are calculated from Lorentzian fit of the spectral lines

After sample characterization, we demonstrate the Zeeman splitting in PL spectroscopy and PL polarization control by magnetic field. Following these, we will discuss the origin of our observations. Figure 2a and b shows the polarization-resolved PL spectra of monolayer and bilayer MoTe_2_ under external magnetic field of −7, 0, and +7 T perpendicular to the sample plane at 2 K. Monolayer PL shows peak *A*_1_ (*B*_1_) with an energy of 1.187 eV (1.164 eV). The bilayer shows emissions at 1.154 and 1.136 eV (peak *A*_2_ and *B*_2_ in Fig. 2b). Peak *A*_2_ (*A*_1_) is attributed to the optical transition of the neutral exciton state in the 2L (1L) MoTe_2_. Peak *B*_2_ (*B*_1_) corresponds to the transition of charged exciton (trion) state in the 2L (1L)^27^. From the figure, we can make two main observations: First, at zero magnetic field, the wavelengths of PL emission are at the same position for *σ*_+_ and *σ*_−_ detection. At −7 T (+7 T), however, the position of the peaks blueshift (redshift) for *σ*_+_ (*σ*_−_) detection, which indicates an energy splitting. Second, the magnitudes of the peaks for *σ*_+_ and *σ*_−_ detection under magnetic field also differ, which manifests itself as magnetic field-dependent PL polarization. Here, the degree of the PL polarization can be defined as ${\eta _{{\rm{PL}}}} = \frac{{{I_{{\sigma _ + }}} - {I_{{\sigma _ - }}}}}{{{I_{{\sigma _ + }}} + {I_{{\sigma _ - }}}}}$, where ${I_{{\sigma _ + }}}$ $\left( {{I_{{\sigma _ - }}}} \right)$ is the intensity of the PL emission in *σ*_+_-out (*σ*_−_-out) configurations.

To further illustrate these results, spectrum splitting and PL polarization for the neutral excitons are quantitatively depicted in Fig. 2c and d, respectively. The Zeeman splitting of an optical transition is fit with Δ*E* = *gμ*_B_ *B*, where *g* is the *g*-factor associated with the magnetic moment in the system, *μ*_B_ is the Bohr magneton, and *B* is the magnetic field. The energy splitting of the exciton state (peak *A*_1_ of 1L and *A*_2_ of 2L) depends linearly on the magnetic field, with slopes of −243 ± 3 and −274 ± 6 μeV T^−1^, corresponding to *g*(*A*_1_) = 4.21 ± 0.06 and *g*(*A*_2_) = 4.73 ± 0.11. Although our experiment shows a finite valley polarization in bilayer MoTe_2_ with near-resonant excitation (Supplementary Note 2; Supplementary Fig. 4), here we focus on PL polarization only with off-resonant excitation. In Fig. 2c and d, the averaged PL polarization of *σ*_+_ and *σ*_−_ excitation is shown, where the PL polarization of the 1L and 2L depends linearly on the magnetic field. We fit the relationship of the PL polarization *η*_PL_ and the magnetic field *B* with *η*_PL_ = *βB*, where *β* is a coefficient with (3.82 ± 0.04) × 10^−2^ T^−1^ for peak *A*_1_ of the 1L and (5.25 ± 0.28) × 10^−2^ T^−1^ for peak *A*_2_ of the 2L. The fit result of the trion (peak *B*_2_) are shown in the Supplementary Fig. 3. In addition, we have measured the temperature dependence of the *g*-factor and PL polarization for both monolayer and bilayer MoTe_2_. As shown in the inset of Fig. 2c and d, although *g*-factor of monolayer exciton stays around 4, the *g*-factor of bilayer varies from 4.73 to 2.54 when the temperature changes from 2 to 70 K. When the temperature increases, PL polarization has a trend to decrease for both monolayer and bilayer as shown in Fig. 2d.

The Zeeman splitting in MoTe_2_ monolayers was already reported, which is attributed to the lifting of the valley degeneracy in the band structure due to the breaking of the time-reversal symmetry in the presence of a magnetic field, so called valley Zeeman splitting, or valley splitting for short^10–13^. The main observation here is that such Zeeman splitting still persists in bilayer, which can not be simply considered as valley Zeeman splitting anymore. Below, we focus on the physical origin of such splittings, as well as the magnetic field-dependent PL polarization.

In monolayer TMDs, the spin and the valley pseudospin are effectively coupled by spin–orbital coupling and broken inversion symmetry^1^. Bilayer TMDs possess another degree of freedom, viz., layer pseudospin^16^. In a bilayer, the Hamiltonian at ±*K*-points can be expressed in a two-band approximation as ${H_{\rm{c}}} = \Delta + {\lambda _{\rm{c}}}{\tau _{\rm{z}}}{s_{\rm{z}}}\sigma _{\rm{z}}^{\rm{c}}$ for the conduction band and ${H_{\rm{v}}} = - {\lambda _{\rm{v}}}{\tau _{\rm{z}}}{s_{\rm{z}}}\sigma _{\rm{z}}^{\rm{v}} + {t_ \bot }\sigma _{\rm{x}}^{\rm{v}}$ for the valence band, where Δ is the bandgap, *λ*_c_ (*λ*_v_) denotes the spin–orbit coupling of conduction (valence) band and *t*_⊥_ is the interlayer coupling of the layers. The strong coupling between the valley (*τ*_z_) and layer $\left( {\sigma _{\rm{z}}^{{\rm{c}},{\rm{v}}}} \right)$ pseudospin, and the real spin (*s*_z_) is a distinguishing feature of bilayers. The layer Pauli operators $\sigma _{\rm{z}}^{\rm{c}}$ ($\sigma _{\rm{z}}^{\rm{v}}$) are expressed in the basis of ${d_{{{{z}}^2}}}$ $\left( {{d_{{x^2} - {y^2}}} \pm i{d_{xy}}} \right)$. The interlayer hopping, *t*_⊥_, vanishes for conduction band due to the symmetry of ${d_{{{{z}}^2}}}$ orbitals. When the spin–orbit coupling strength *λ*_v_ is much larger than the interlayer hopping amplitude, holes are primarily confined to either upper or lower layer, which can be labeled with layer pseudospin up $\left| u \right\rangle$ or down $\left| l \right\rangle$.

**Fig. 3:**
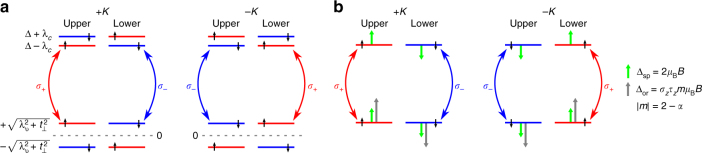
Origin for Zeeman splitting in bilayer. **a** Single-particle energy states at ±*K*-points of bilayer TMD at zero magnetic field and in presence of interlayer hopping *t*_⊥_. The spin–valley-layer locking results in optical selection rules such that in both valleys, the spin degree of freedom is locked to the emission helicity. **b** Schematic diagram of the Zeeman splitting in bilayer MoTe_2_ under positive magnetic field at ±*K*-points. Red (blue) transition indicates the PL emission with photon energy *E*_+_ (*E*_−_) and circular polarization *σ*_+_ (*σ*_−_). The upper and the lower layer in the same valley have opposite spin. Green and gray arrows show the magnetic moment contributions of the spin and atomic orbital to the Zeeman splitting, respectively. In the presence of a positive magnetic field, the spin contributions cancel, whereas the intracellular or atomic orbital contribution leads to *E*_+_ < *E*_−_ in both the valleys

Figure 3a depicts the energy level diagram at zero magnetic field emphasizing the spin–valley-layer locking in bilayer. At a given energy in a given valley, different layers carry opposite spins. The lowest energy single-particle optical transitions giving rise to excitonic resonance for different valley, layer, and spins are also shown in Fig. 3a. As the spin is conserved in the optical transition (singlet exciton), spin–valley-layer locking leads to emission helicity being locked with the spin degree of freedom in both valleys. Upon diagonalizing *H*_v_, the hole energies shift from ±*λ*_v_ to $\pm \sqrt {\lambda _{\rm{v}}^2 + t_ \bot ^2}$ and the new eigenstates are admixtures of *d* ^*u*^ ± *id* ^*u*^ and *d* ^*l*^ ∓ *id* ^*l*^ orbitals. Unlike the case of monolayer where helicity of emission is tied to the valley degree of freedom, optical transitions of either helicity are present in both valleys for bilayers. In the absence of magnetic field, all four optical transition depicted in Fig. 3a are degenerate.

When an out-of-plane *B*-field is applied, conduction and valence band energy will be shifted, in accordance with the respective magnetic moments as shown in Fig. 3b. The conduction band states have contribution only from the spin as $d_{{z}}^2$ orbitals do not carry any magnetic moment, whereas the valence band states have orbital magnetic moment (intracellular contribution) stemming from *d* ± *id* orbitals in addition to the spin contribution. In the ideal case without substrate effect, spatial inversion symmetry is restored for bilayers^28^, which makes intercellular contribution vanish. With possible substrate effect^29^, there can still be asymmetry in bilayer, which might still introduce the intercellular term. The spin Zeeman shift can be written as Δ_*s*_ = 2*s*_z_ *μ*_B_ *B*. As Δ_*s*_ has the same value for conduction and valence band, it thus does not contribute to the net energy shift. Thus, intracellular contribution which, differs for the two bands, causes a measurable shift in the optical transition energies.

In the limit of negligible interlayer coupling, the valence band is mainly comprised of *d* ± *id* orbitals with *m* = ±2, whereas the conduction band has *m* = 0. This intracellular contribution leads to a valley Zeeman splitting with a *g*-factor of 4 in monolayer TMDs. The bilayer case is in stark contrast with this as can be seen from Fig. 3b—although the valley degeneracy is not lifted, each valley experiences a splitting of emission helicity (*σ*_+_/*σ*_−_) due to intracellular contribution. In other words, whereas there is a lifting of degeneracy of *σ*_+_/*σ*_−_ emission in bilayers in the presence of *B*-field, it does not imply a valley Zeeman splitting as the emission helicity is no longer tied to the valley degree of freedom. Instead, the helicity of emission is tied to the spin degree of freedom. A *g*-factor of 4 is thus expected for bilayer Zeeman splitting as well, however, due to finite interlayer hopping, the valence band states are no longer purely *d* + *id* or *d* − *id* but an admixture of the two.

The exact eigenstates of *H*_v_ at *K*-valley (*τ*_z_ = 1) and spin up (*s*_z_ = 1) are given by *u*_+_ = (cos *θ*/2, sin *θ*/2)^*T*^ and *u*_−_ = (sin *θ*/2, cos *θ*/2)^*T*^ in the basis of where ${\rm{cos}}\,\theta = \frac{\lambda }{{\sqrt {{\lambda ^2} + t_ \bot ^2} }}$. Thus, the magnetic moment of valence band states reduces from *m* = ±2 to $\tilde m = \left( { \pm 2} \right){\rm{co}}{{\rm{s}}^2}\theta {\rm{/}}2 + \left( { \mp 2} \right){\rm{si}}{{\rm{n}}^2}\theta {\rm{/}}2$ = $\pm 2\,{\rm{cos}}\,\theta = \pm 2\frac{\lambda }{{\sqrt {{\lambda ^2} + t_ \bot ^2} }}$. This would imply a Zeeman splitting *g*-factor of $\frac{{4\lambda }}{{\sqrt {{\lambda ^2} + t_ \bot ^2} }}$. From recent report of *A* − *B* splitting of monolayers, we get *λ* of ~135 meV^30, 31^. Assuming *B* exciton has the same energy for monolayer and bilayers^31^, and the difference of exciton peak position is $2\sqrt {{\lambda ^2} + t_ \bot ^2} - 2\lambda = 33\,{\rm{meV}}$, we get interlayer coupling of *t*_⊥_ = 69 meV and *g*-factor of 3.56. The difference between the predicted value and experimental value of g factors might come from several origins. First, it can come from intercellular components which comes from inversion symmetry breaking due to substrate effect. In addition, the intracellular contribution from other orbitals (e.g., *p*-orbitals for the conduction band) will need to be considered to calculate the value of precise g-factor^35^. We note that change of *g*-factor for bilayer is much larger than the case of monolayer. We speculate that the temperature dependence for bilayer arises due to the change in the interlayer distance with temperature, just as lattice constant changes with temperature^32–34^. A systematic understanding of the temperature dependence of *g*-factor is very interesting in its own right and is left for future investigations.

Finally, we discuss the magnetic field dependence of *η*_PL_ shown in Fig. 2d. As the PL polarization is primarily independent of the excitation polarization, we can conclude that there is fast-spin relaxation, which leads to creation of both *σ*_+_ and *σ*_−_ excitons upon excitation. At zero field, conversion of *σ*_+_ to *σ*_−_ and vice versa is equally likely leading to emission from both helicities, as dictated by time-reversal symmetry. At finite *B*-field, the emission intensity of the lower energy peak is always larger. This is true even when the polarity of the *B*-field is reversed implying that the higher energy exciton is transformed into the lower energy exciton with the opposite emission helicity on a timescale, which is comparable to exciton lifetime.

**Fig. 4:**
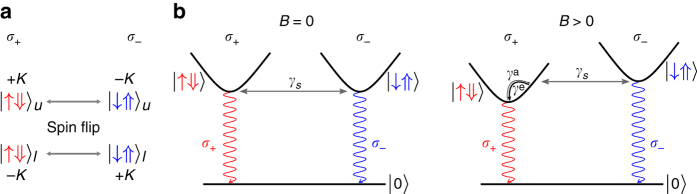
Origin for PL polarization in bilayer. **a** The requirement for the switching of light helicity can be thought of as interconversion between circularly polarized excitons, $\sigma _ + ^{{\rm{ex}}}$ and $\sigma _ - ^{{\rm{ex}}}$. The emission helicity is tied to the spin of the electron in the exciton, which is formed out of electron–hole pairs in both valleys/layers. The hole spin is labeled by thick vertical arrows and in our convention have spin, which is opposite to that of the electron. Owing to spin–valley-layer locking, both spin and valley need to be flipped for the switching of helicity, in the absence of interlayer hopping. **b** At positive magnetic field, the selective conversion of $\sigma _ - ^{{\rm{ex}}}$ to $\sigma _ + ^{{\rm{ex}}}$ is energetically favorable due to the Zeeman splitting

If we assume that the interlayer coupling is suppressed due to large spin–orbit coupling, the conversion of a *σ*_+_-exciton to a *σ*_−_-exciton and vice versa requires flipping of both spin and valley degrees of freedom, as shown in Fig. 4a. The spin angular momentum required for such a process is possibly provided during scattering with residual charge carriers present in the sample due to accidental doping. Although at zero *B*-field, such a spin flip-induced conversion of exciton helicity can occur in both directions, at finite *B*-field, conversion to the lower energy exciton is energetically favorable. To explain the dependence of *η*_PL_ with *B*-field, we assume that the spin-flip process is energy conserving, whereas the energy relaxation via phonons primarily conserves spin. Although spin flip via phonon is possible in presence of spin–orbit coupling, it is usually slower than spin-conserving processes^36^. As shown in Fig. 4b, at finite field, spin flip can happen from the higher energy exciton to the excited states of lower energy exciton band at the same energy, which then relax to the lowest energy states by phonons. The reverse process must first involve phonon absorption followed by spin flip due to the absence of opposite spin states for the lowest energy exciton. As the phonon absorption is suppressed by the Boltzmann factor, exp(−Δ_B_/*k*_B_ *T*) for a Zeeman splitting of Δ_B_, the intensity of PL from the lowest energy exciton is dominant. The quantitative dependence of *η*_PL_ on *B* depends on the spin-flip rate *γ*_s_, exciton lifetime *γ*_l_ and the phonon relaxation rate *γ*_ph_ which appear to be comparable to each other in bilayer TMDs (Supplementary Note 3; Supplementary Fig. 5).

## Discussion

In summary, we have experimentally demonstrated the Zeeman splitting in bilayer TMDs and discussed their origin from spin–valley-layer coupling. Electrical control of orbital magnetic momentum as demonstrated previously^2, 20^, together with magnetic control here will form a complete toolbox set for controlling valley and layer pseudospins. Magnetoelectric effect by the interference between electrical and magnetic field will be naturally the next step towards quantum gates or quantum entanglement between spin, valley, and layer degree of freedom in bilayer platforms^16^. Optical stark effect by means of pseudomagnetic field has been demonstrated to control the coherence of valley pseudospins^37–39^. Real magnetic control of bilayer as demonstrated here, combined with pseudomagnetic method provides access to manipulate the coherence in the bilayer system.

## Methods

### Spectroscopy experiment setup

The Raman spectra are taken at room temperature with an excitation wavelength of 532 nm using a commercial WITech confocal Raman spectrometer. We use a homemade fiber-based confocal microscope for polarization-resolved PL spectroscopy. The wavelength of the excitation is 795 nm (1.560 eV) for off-resonant excitation and 1040 nm (1.192 eV) for near-resonant excitation. Polarizers and quarter-wave plates are installed on the excitation and detection arm of the confocal microscope for polarization-selective excitation and PL detection. The PL emission is directed by an multi-mode optical fiber into a spectrometer (Princeton Instruments) with a liquid nitrogen-cooled infrared camera for spectroscopy recording. The sample is loaded into a magneto-cryostat (Cryomagnetics close-cycle cryostat (CMag) for off-resonant experiment and Quantum Design Physical Properties Measurement System (PPMS) for near-resonant experiment) and cooled down to 2–4 K. The magnetic field is applied perpendicular to the sample plane ranging from −7 to +7 T (CMag) or −9 to +9 T (PPMS).

### Preparation of MoTe_2_ thin flakes

The MoTe_2_ single crystals are synthesized through chemical vapor transport using iodine as the transport agent. A scotch tape-based mechanical exfoliation method is used to peel thin flakes from bulk crystal onto degenerately doped silicon wafer covered with a layer of 285 nm thermally grown silicon dioxide. Optical microscopy (Olympus BX-51) is used to identify thin flake samples with different thickness via optical contrast.

### Data availability

The data that support the findings of this study are available from the corresponding authors on request.

## Electronic supplementary material

Supplementary Information
