# Supplementary material for: Zeeman splitting via spin-valley-layer coupling in bilayer MoTe2
Source: Nat Commun. 2017 Oct 6;8:802. doi: 10.1038/s41467-017-00927-4 (PMC5630581; doi:10.1038/s41467-017-00927-4)
Supplement: Supplementary file 1 — Supplementary Information [file 41467_2017_927_MOESM1_ESM.pdf]

Supplementary Note 1. Sample characterization and Zeeman splitting in bilayer $MoTe_2$

Normalized temperature-dependent PL for both monolayer and bilayer $MoTe_2$ is shown in Supplementary Figure 1. The intensity of the A_2 peak is linearly dependent on the excitation power (Supplementary Figure 2), which indicates its exciton character. Peak B_2 with an energy lower than peak A_2 is attributed to trion peak. Zeeman splitting and PL polarization of the trion state of the bilayer are shown in Supplementary Figure 3.

Supplementary Note 2. Valley polarization in bilayer $MoTe_2$

The magneto-photoluminescence (PL) spectra of the bilayer $MoTe_2$ near-resonantly pumped with σ_+ and σ_- polarization are shown in Supplementary Figure 4 a and b. For bilayer, although the emission polarization is predominantly tuned by the external magnetic field and nearly independent of the polarization of the excitation, there is in fact a finite correlation between the excitation and emission polarization. At 0T the PL intensity of the co-polarization configuration is larger than that of the cross-polarization configuration (see Supplementary Figure 4 a and b). In Supplementary Figure 4 c and d, the degree of polarization is not zero-crossing. The co-polarization (cross-polarization) configured curve offsets positively (negatively), which exhibits a polarization-selecting behavior. In the monolayer reference flake there is no such polarization-selective offset (black symbols and lines in Supplementary Figure 4 d. Such polarization-selective correlation, so called *Valley polarization*, has also been observed in bilayer WS_2[1].

Supplementary Note 3. Model to explain the magnetic field dependence of PL polarization

As discussed in the main text, we assume that interlayer coupling is suppressed due to large spin-orbit coupling. Below we use a rate equation model to estimate the decay rates. We put the rate of optical pumping to both σ_+ and σ_- excitons is the same, Γ_{pump}. Let the energy conserving spin flip rate be γ_s, the phonon emission rate be γ_e while the phonon absorption rate be γ_a. Assuming a lattice temperature of T, we have $\gamma_a = \gamma_e e^{\frac{-g\mu_B B}{k_B T}}$. Furthermore, we assume γ_l, the total lifetime of both σ_+ and σ_- excitons to be the same. As the spin-flip process is assumed to be energy conserving, we can work with three levels to describe the conversion process between σ_+ and σ_- exciton. Let n_1 (n_3) be the population in the higher (lower) energy exciton and n_2 be

population in the state obtained after elastic spin-flip from the higher energy exciton.

The following set of rate equations relate the steady populations

$$\Gamma_{pump} - (\gamma_s + \gamma_l)n_1 + \gamma_s n_2 = 0 \tag{1}$$

$$\gamma_s n_1 - (\gamma_e + \gamma_s)n_2 + \gamma_a n_3 = 0 \tag{2}$$

$$\Gamma_{pump} + \gamma_e n_2 - (\gamma_l + \gamma_a)n_3 = 0. \tag{3}$$

By solving the equations above, we obtained the following equation for PL polarization, η_{PL}

$$\eta_{PL} = sgn(B)\frac{1 - e^{\frac{-g\mu_B|B|}{k_BT}}}{1 + e^{\frac{-g\mu_B|B|}{k_BT}} + A_0}, \tag{4}$$

where $A_0 = \gamma_l/\gamma_e + \gamma_l/\gamma_s$.

This model is used to fit the experimental data of exciton and trion PL polarization in both monolayer and bilayer $MoTe_2$. Supplementary Figure 5 shows the fitting result under different sample temperature. The lattice temperature is assumed to be the same as sample temperature except for sample temperature = 2 K where a higher lattice temperature = 10 K is needed for the data fitting. This might come from sample heating due to optical excitation, but its origin needs further investigation. The value of g factor is obtained from the valley splitting data while A_0 is used as fitting parameter.

Based on Supplementary Figure 5, it can be said that our simple model qualitatively captures the magnetic field dependence of PL polarization data reasonably well. Additionally, the temperature dependence of the decay rates can analyzed from the fitting result. As can be seen from Supplementary Figure 5, A_0 has a tendency to decrease as the lattice temperature increases. This can be attributed to the increasing contribution of the spin-flipping process involving longitudinal phonon [2] at higher temperature which causes the scattering rate γ_s increases with temperature.

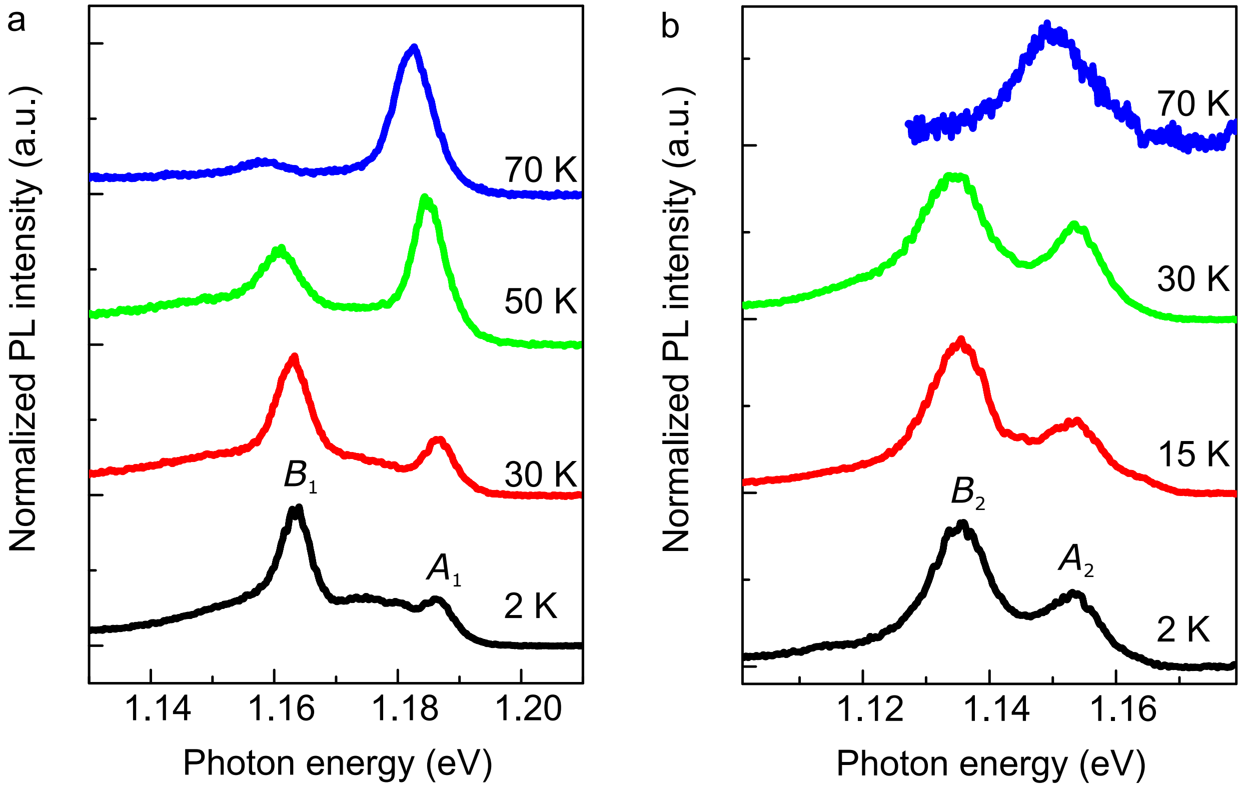

Supplementary Figure 1: **Normalized temperature-dependent PL spectra**. **a**, 1L $MoTe_2$. **b**, 2L $MoTe_2$. The PL spectra are taken with 1.560 eV (795 nm) excitation energy. The exciton and trion state emission are labeled as A_1 (A_2) and B_1 (B_2) for 1L (2L) $MoTe_2$. The curves are offset for clarity. At 70K, the emission of the trion state vanishes.

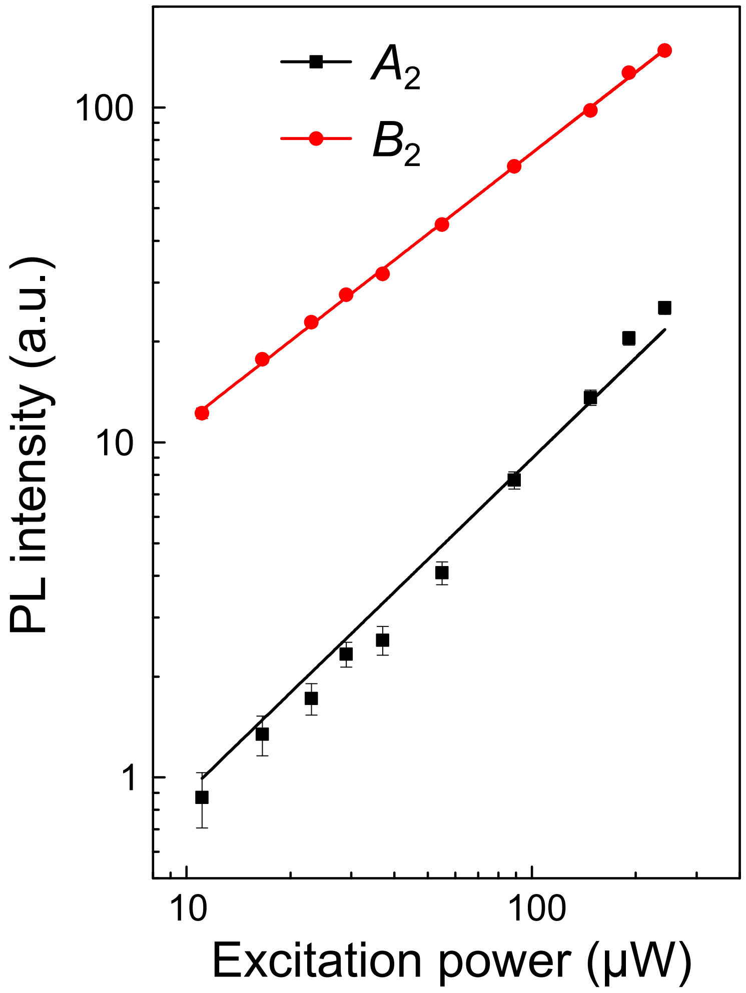

Supplementary Figure 2: **PL intensity of exciton and trion emission as a function of the excitation power in 2L** $\mathbf{MoTe_2}$. The exciton (Peak A_2, black) exhibits a linear power dependence, whilst the trion (Peak B_2, red) shows a sublinear dependence with $I_{PL} \propto I_{ex}^{0.8}$, where I_{PL} and I_{ex} are the intensity of the excitation and photoluminescence, respectively. The error bars are calculated from Lorentzian fit of the spectral lines.

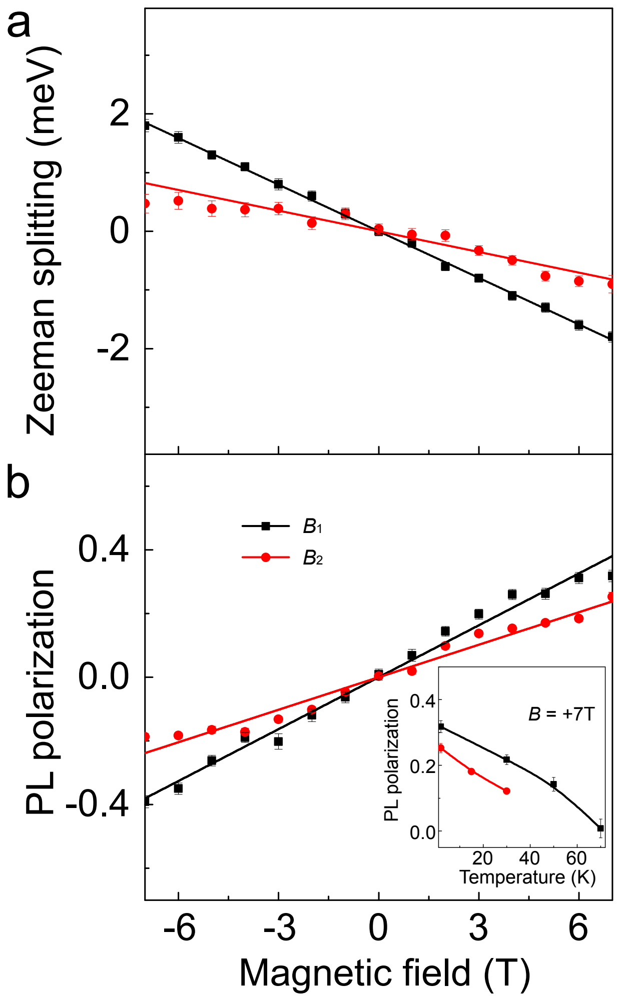

Supplementary Figure 3: **Trion emission of 1L and 2L** $\mathbf{MoTe_2}$ **in magnetic field**. **a**, valley Zeeman splitting **b**, PL polarization, in 2L (red symbols and line) $MoTe_2$. The inset shows the temperature dependence of the PL polarization in 2L (red symbols and line). The symbols are experimental data. The solid lines are the linear regression of the experimental data. The black symbols and lines are data of the 1L $MoTe_2$ for reference. The error bars are calculated from Lorentzian fit of the spectral lines.

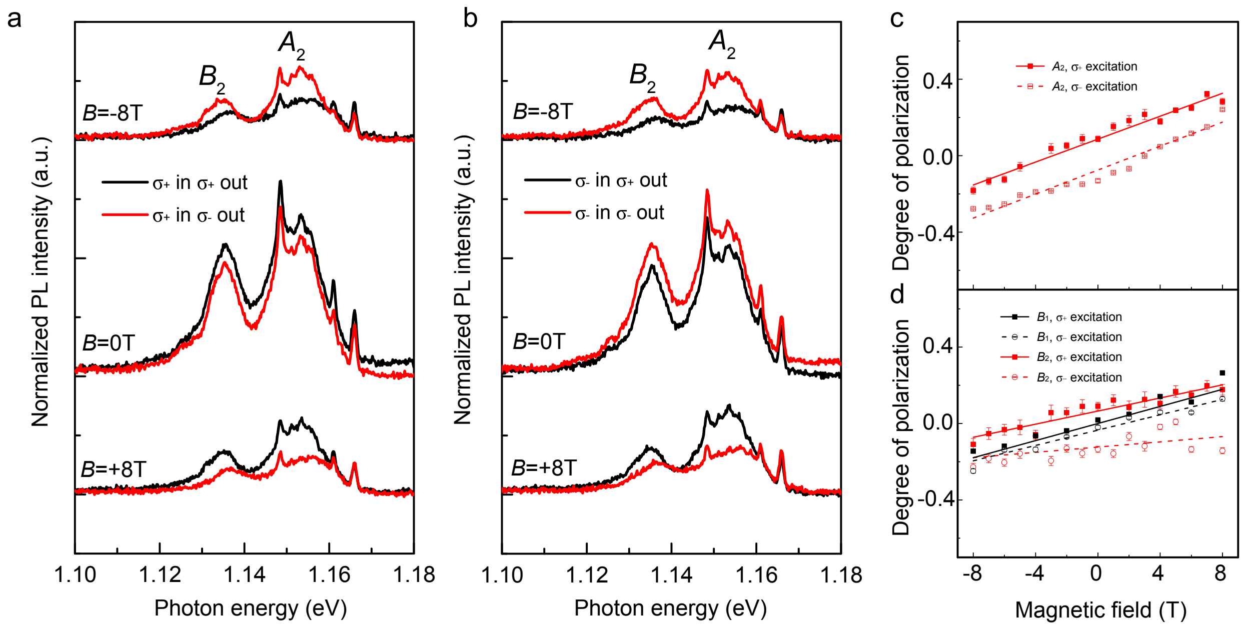

Supplementary Figure 4: **Near-resonant magneto-photoluminescence. a-b**, Polarization-resolved PL spectra of 2L $MoTe_2$ excited by σ_+ (**a**) and σ_- (**b**) polarization taken at -8 T, 0 T and +8 T at 4.2 K with an excitation energy of 1.192 eV (1040 nm). The excitation is set to σ_+ circular polarization. The detection polarization is configured to σ_+ (black solid lines) and σ_- (red solid lines) circular polarization. Here the PL spectra are normalized for each particular magnetic field and offset for clarity. Sharp peaks in the bilayer PL spectra are Raman scattering. **c**, PL polarization extracted from the exciton emission (Peak A_2) in 2L $MoTe_2$. The symbols are experimental data and lines are fitting results. **d**, PL polarization extracted from the Trion emission in monolayer $MoTe_2$ (Peak B_1) and bilayer $MoTe_2$ (Peak B_2). The error bars are calculated from Lorentzian fit of the spectral lines.

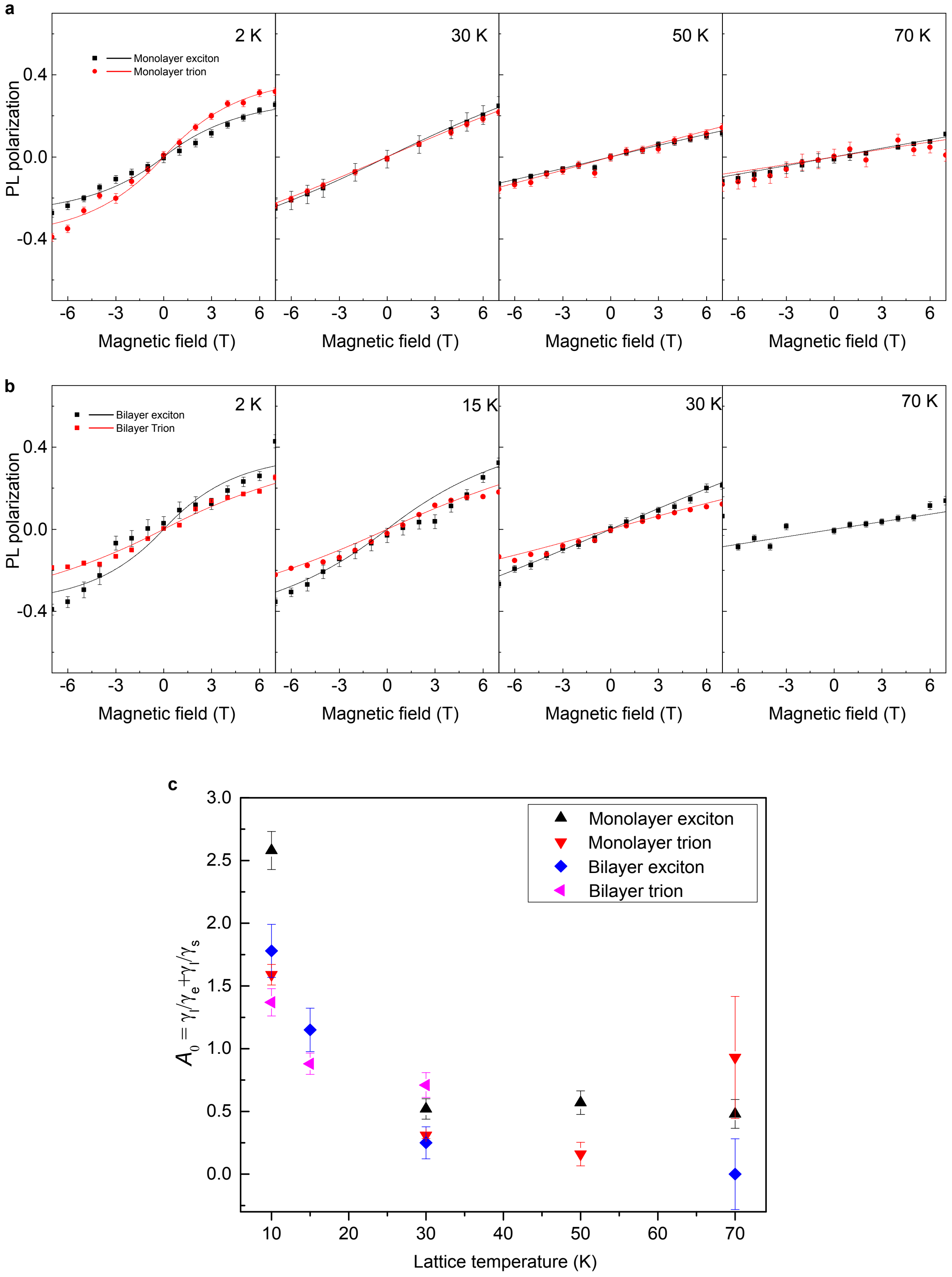

Supplementary Figure 5: **PL polarization fitting**. **a-b**, The data and the fitting result of the PL polarization for the monolayer $MoTe_2$ (**a**) and for the bilayer $MoTe_2$ (**b**). In both cases, the model described in the Supplementary Note is used to do the fitting. (**c**), The value of the fitting parameter A_0 is shown as a function of the lattice temperature. The error bars are calculated from Lorentzian fit of the spectral lines.

Supplementary References

[1] Zhu, B., Zeng, H., Dai, J., Gong, Z., and Cui, X. (2014). Anomalously robust valley polarization and valley coherence in bilayer WS2. *Proceedings of the National Academy of Sciences*, **111**, 11606-11611.

[2] Kioseoglou, G., Hanbicki, A. T., Currie, M., Friedman, A. L., and Jonker, B. T. (2016). Optical polarization and intervalley scattering in single layers of MoS_2 and $MoSe_2$. .*Scientific reports*, **6**, 25041.
